# Crosstalk between PTEN/PI3K/Akt Signalling and DNA Damage in the Oocyte: Implications for Primordial Follicle Activation, Oocyte Quality and Ageing

**DOI:** 10.3390/cells9010200

**Published:** 2020-01-14

**Authors:** Mila Maidarti, Richard A. Anderson, Evelyn E. Telfer

**Affiliations:** 1 MRC Centre for Reproductive Health, Queens Medical Research Institute, University of Edinburgh, Edinburgh EH16 4TJ, UK; mila.maidarti@ed.ac.uk (M.M.); richard.anderson@ed.ac.uk (R.A.A.); 2 Institute of Cell Biology, University of Edinburgh, Edinburgh EH9 3FF, UK; 3 Obstetrics and Gynaecology Department, Faculty of Medicine, Universitas Indonesia, Jakarta 10430, Indonesia

**Keywords:** PTEN/PI3K/Akt, follicle activation, DNA damage response (DDR), ageing

## Abstract

The preservation of genome integrity in the mammalian female germline from primordial follicle arrest to activation of growth to oocyte maturation is fundamental to ensure reproductive success. As oocytes are formed before birth and may remain dormant for many years, it is essential that defence mechanisms are monitored and well maintained. The phosphatase and tensin homolog of chromosome 10 (PTEN)/phosphatidylinositol 3-kinase (PI3K)/protein kinase B (PKB, Akt) is a major signalling pathway governing primordial follicle recruitment and growth. This pathway also contributes to cell growth, survival and metabolism, and to the maintenance of genomic integrity. Accelerated primordial follicle activation through this pathway may result in a compromised DNA damage response (DDR). Additionally, the distinct DDR mechanisms in oocytes may become less efficient with ageing. This review considers DNA damage surveillance mechanisms and their links to the PTEN/PI3K/Akt signalling pathway, impacting on the DDR during growth activation of primordial follicles, and in ovarian ageing. Targeting DDR mechanisms within oocytes may be of value in developing techniques to protect ovaries against chemotherapy and in advancing clinical approaches to regulate primordial follicle activation.

## 1. Introduction

In mammalian females, oocytes are formed before birth and are surrounded by somatic cells (granulosa cells) to form structures known as follicles. Oocytes have entered meiosis and are arrested at the dictyate stage of prophase I with the most immature stage (primordial follicles) forming the store of female germ cells that will be utilised throughout reproductive life (reviewed in [1]). The pool of primordial follicles is progressively reduced with age leading to reproductive senescence [2,3,4]. Follicles are gradually lost from the pool either through death or by activation of the growth pathway. Therefore the rates of activation and degeneration determine the size of the pool and the time to onset of menopause [5]. Once follicles are recruited into the growing pool, pre-granulosa cells differentiate to form a single layer of cuboidal cells surrounding the oocyte. In parallel, the oocyte increases in size and undergoes further growth and maturation whilst still being maintained in meiotic arrest. These processes are referred to as primordial follicle activation [6]. Primordial follicles may be quiescent for many years and in humans for several decades, highlighting the importance of potential DNA damage accumulation [7] that may threaten genomic integrity. In this context, a robust surveillance mechanism is essential to ensure that oocytes with DNA damage have it repaired or are eliminated with prevention of further growth and development [8,9], thus maintaining the quality of oocyte and any resulting embryo throughout the reproductive lifespan [10].

The phosphatase and tensin homolog of chromosome 10 (PTEN)/phosphatidylinositol 3-kinase (PI3K)/protein kinase B (PKB, Akt) pathway is one of the major non-gonadotropic insulin signalling pathways that coordinates the activation, growth and differentiation of follicles [6,11]. The pathway functions to control a myriad of cellular functions involving cell metabolism, proliferation and survival [12,13]. There is evidence to support the existence of crosstalk between the PI3K/Akt signalling pathway and the DNA damage response (DDR) in cells [14,15,16], indicating the importance of the consequences of interference with one of these pathways on the other. High PI3K/Akt activity is linked to a decline in the number of primordial follicles and ovarian ageing [17,18]. Ovarian ageing is associated with impaired DDR within oocytes [19,20,21,22] and this can also be induced following exposure to DNA damaging agents [23,24,25,26,27,28]. Nevertheless, taking advantage of PI3K/Akt signalling pathway effects on follicular recruitment, PTEN inhibition, as a central negative regulator of the pathway, has been widely used to activate primordial follicles in a range of species [18,29,30,31,32,33]. Most importantly, pregnancies have been achieved in women following transplantation of small fragments of ovarian cortex after exposure to pharmacological inhibitors of PTEN [34]. Recent studies suggest that activation of follicles by these methods may be damaging to subsequent growth and survival of follicles [35,36,37], indicating that further investigation is required to fully understand the impact and implications of follicle activation using pharmacological manipulation of this pathway.

A great deal of evidence, discussed below, suggests that the PI3K/PTEN/Akt pathway is essential in regulating cell-cycle checkpoint initiation and DNA repair and that the lack of PTEN in cells may cause genomic instability [38,39]. The ability to respond to such damage is crucial to ensure primordial follicle survival and to support the production of mature oocytes with a minimised risk of meiotic abnormalities against the adverse effects of age (reviewed in [40]) and ultimately to maintain reproductive lifespan. Surveillance mechanisms within oocytes to ameliorate DNA damage are essential as, during reproductive life, oocytes (and granulosa cells) can be subjected to DNA damage: this mainly occurs in the long-lived primordial follicles as a consequence of external and internal insults [21]. DNA double strand-breaks (DSBs) do not occur as frequently as other lesions, but persistent unrepaired DNA DSBs are the most severe type of damage and may lead to genomic instability [21,41,42,43].

In this review, we will limit the discussion to the DNA damage/DSBs repair pathway, and primarily focus on the mechanisms used by oocytes within primordial follicles to protect themselves against DNA damage throughout their lifespan. The DDR mechanism in granulosa cells will be discussed where relevant. Crosstalk between the PI3K/PTEN/Akt pathway and DDR has been linked to increased DNA damage and impaired DNA repair protein interactions in ovarian follicles activated in vitro [29]. Such findings will be important in elucidating the impact of pharmacological activation of primordial follicles by manipulation of the PI3K/PTEN/Akt pathway and its impact on DDR.

## 2. Methods

**Figure 1:**
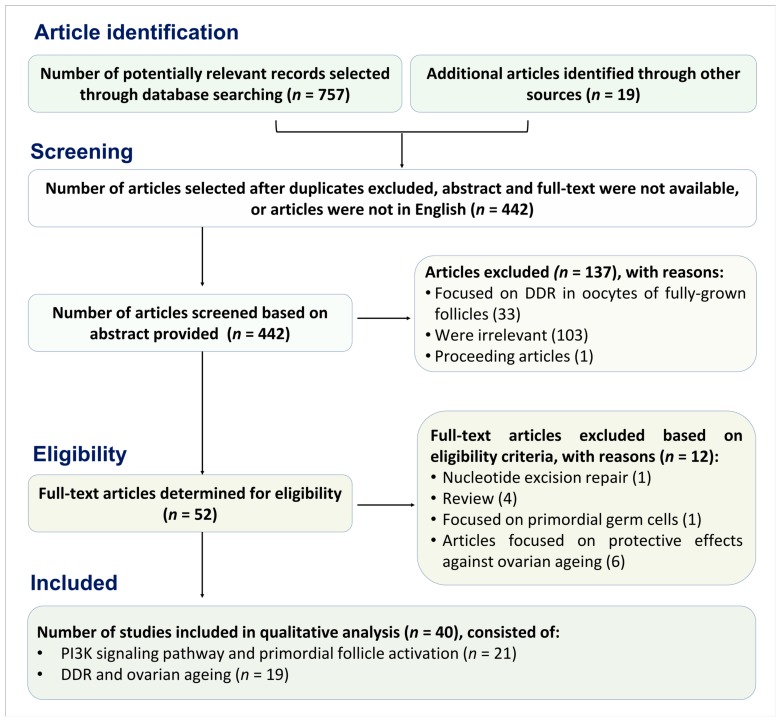
Flow chart following Preferred Reporting Items for Systematic Reviews and Meta-Analyses (PRISMA) guidelines to determine the study included into qualitative analysis.

Published articles including original research, peer-reviewed and reviews were searched systematically in PubMed (Medline) database using specific terms such as ‘DNA damage’, ‘oocytes’, ‘primordial follicle activation’, ‘PI3K/Akt’, ‘ovarian ageing’ and ‘chemotherapy’. Abstracts and conferences proceeding were not included. The search yielded 757 relevant references of English language literature. Article selection was conducted using Preferred Reporting Items for Systematic Reviews and Meta-Analyses (PRISMA) guidelines [44]. The references in these articles were searched manually to retrieve additional articles, and an additional 19 articles were included. These were then screened for duplication, to ensure only articles related to DNA damage repair mechanism in primordial follicles were included. Only original research articles meeting the following eligibility criteria were included in the final search results: original research articles published from 1990 to 2019, full articles available, articles in English and not symposia proceeding. Manuscripts were selected concerning primordial follicle activation in association with PI3K signalling pathway, ovarian ageing and DDR in oocytes of immature follicles. A total of 52 published full-text articles were included after cross-referencing, and 40 articles were analysed qualitatively (Figure 1).

## 3. DNA Damage Repair Pathway within Primordial Follicles

The DNA of a cell is continuously threatened by various types of damage that may cause a reduction in cellular function, cell cycle progression and DNA repair [45]. Exogenous sources of DNA damage include environmental agents such as ultraviolet, radiation and chemotherapeutic drugs [24]. Reactive oxygen species (ROS) are also an endogenous source of damage within somatic cells [46] and oocytes [20,47]. DNA damage constitutes a significant issue in non-dividing or slowly dividing cells as a large amount of DNA damage may accumulate over time. Any damage that does not cause cell cycle arrest will tend to induce replication errors leading to mutations [20]. However, all cells are endowed with the capacity to ameliorate the threats to DNA, which occurs mainly at the G1/S and G2/M-phase transition. Cells with DNA damage respond in various ways to activate an appropriate DDR pathway. A mild injury may not result in serious consequences as it can be repaired directly without cell cycle arrest. While severe DNA damage may result in cell cycle arrest, allowing sufficient time to repair DNA damage. During this time, a sequence of DDR proteins is activated and the cell’s fate depends on its capacity to repair the damage [48].

DNA DSBs can be repaired by two main mechanisms: non-homologous end-joining (NHEJ) [49], and homologous recombination (HR) [20,50]. NHEJ is error-prone since it mediates the direct re-ligation of the two ends of broken DNA and is not based on a complementary DNA template. Given its error-prone nature, NHEJ is commonly accompanied by deletion or insertion of base pairs [51,52]. NHEJ primarily occurs at the G0/G1 phase [53] and can be independent of the cell cycle [54]. NHEJ is the most common type of DNA damage repair in mitotic cells. In contrast, HR is largely error-free and is functionally dominant at S and G2/M phases of the cell cycle when sister chromatids are available as a template for accurate DNA repair [55]. In this context, HR is the primary mode of DNA DSBs repair in meiotic cells. Both pathways are evident and can be functionally active in mammalian oocytes [56,57], although HR predominates in oocytes at all stages of development [10,48,58,59,60,61,62]. Given that primordial follicles are arrested at G2/M and accurate repair is a prerequisite to conserve genetic information [63], HR appears to be the pathway of choice for oocytes within primordial (immature) follicles [10,48,58,59]. While NHEJ can occur in the late stage of oocyte development [49,60,64].

HR requires the recognition of the DNA DSBs by the meiotic recombination 11 (MRE11)-Rad50-nijmegen breakage syndrome 1 (NBS1) (MRN) complex. The binding of MRN complex to DSB free ends allows the NBS1 protein to interact with ataxia telangiectasia mutated (ATM) dimers leading to autophosphorylation of ATM at a serine residue (367, 1893 and 1981) [65]. Detection of DNA damage attracts ATM kinase to the DNA DSB sites, through direct interaction between ATM and the C-terminal region of NBS1 [66]. It has been reported that ovaries from MRE11 mutant mice showed a marked increase in unrepaired DSBs, with primordial follicle loss and infertility although the number of mature follicles did not differ between wild type and mutant mice [67]. However, meiotic progression in mutant mice was delayed with only 5% of oocytes being able to complete synapsis [67], suggesting a key role of MRE11 in oocyte DDR.

**Figure 2:**
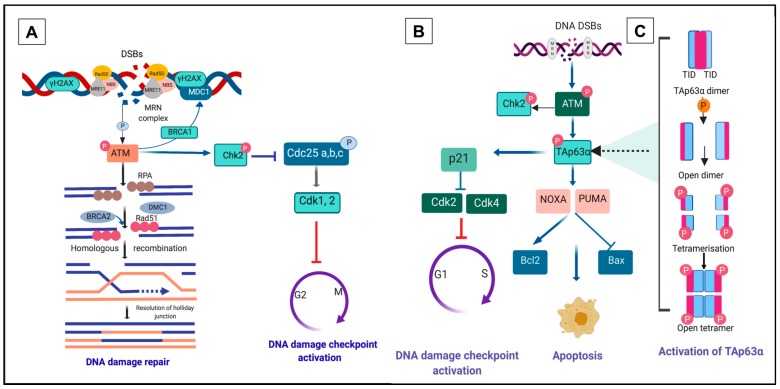
DNA double-strand breaks (DSBs) response pathway. (**A**) Homologous recombination (HR) repair pathway to combat DNA DSBs. Detection and recognition of DNA DSBs by the meiotic recombination 11-Rad50-nijmegen breakage syndrome 1 (MRN) complex (MRE11-RAD50-NBS1) triggers phosphorylation of ataxia telangiectasia mutated (ATM). Activation of ATM results in the phosphorylation of several DNA damage response (DDR) kinases such as histone protein, H2A variant, H2AX, at Serine 139 to generate γH2AX, checkpoint kinase 2 (Chk2) and p53 (TAp63α in primordial oocytes), mediating the effects of ATM on DNA damage repair, cell-cycle arrest and apoptosis. p63 induces cell-cycle arrest by activating the transcription of p21, which may hinder cell cycle progression through inhibition of cyclin-dependent kinase 2 (Cdk2) and Cdk 4 activity. Mediator DNA damage checkpoint protein 1 (MDC1) binds to γH2AX via breast cancer susceptibility gene 1 (*BRCA1*) and forms foci that co-localise with γH2AX. In oocytes, the DNA strand resection is activated and leads to homologous recombination (HR). Activation of HR generates single-strand DNA (ssDNA) at multiple steps and requires a specific factor, replication protein A (RPA). The ssDNA binding protein complex RPA in oocytes is replaced by Rad51 and meiotic cDNA1 (Dmc1). (**B**) Activated Chk2 promotes degradation of cell division cycle (Cdc25) and ultimately provokes cell cycle arrest through phosphorylation of Cdk2 and 4. Alternatively, in response to excessive or irreparable DNA damage, p63 may induce a cascade of apoptotic signalling pathway that requires transcriptional induction of p53 upregulated modulator of apoptosis (PUMA) and NOXA [24,92]. Apoptosis is controlled by the balance between pro-apoptosis B-cell lymphoma 2 (Bcl2) and anti-apoptosis B-cell lymphoma (Bcl)-associated X (BAX) activity. (**C**) An interplay of dimeric to the tetrameric formation of TAp63α. Phosphorylation of TAp63α ultimately transforms the inactive dimeric form of TAp63α to the active tetrameric form (figure adapted from [84,85,87]).

ATM in turn phosphorylates a specific histone protein, H2AX, at the C-terminal serine 139 to generate γH2AX, which binds specifically to the DNA damage sites and controls the recruitment of DNA repair proteins. The critical role of ATM in DDR is demonstrated by a study using mouse ovaries in culture exposed to phosphoramide mustard (PM), a metabolite of cyclophosphamide (CP). Increased γH2AX in oocytes occurred 24 h after exposure and consequently induced substantial follicle loss. Interestingly, the administration of the ATM inhibitor KU55933, reduced the adverse impact of PM on follicle depletion, emphasising the importance of ATM in the DDR [68]. Phosphorylation of γH2AX initiates a downstream pathway resulting in DNA repair or cell cycle arrest (reviewed in [20]). γH2AX is extensively phosphorylated from minutes to hours following the detection of DNA breaks, quantitatively reflecting the severity of the damage [69,70,71]. Mediator DNA damage checkpoint protein (MDC1) is then activated and bound to γH2AX, mediated by breast cancer susceptibility gene 1 (*BRCA1*; Figure 2A). MDC1 forms foci that co-localise with γH2AX within minutes after the damage occurs and provides positive feedback, recruiting additional MRN complexes and thus leading to propagation of γH2AX at sites of DNA breaks [72].

Phosphorylation of ATM is the first step in the initiation of G2 checkpoint activation in the DNA damage repair pathway [73]. Activation of ATM upregulates downstream pathways leading to effective DNA repair through HR/NHEJ (HR in oocytes of primordial follicle, Figure 2A), initiation of checkpoint kinase 2 (Chk2, Figure 2A) or apoptosis (through activation of TAp63α, Figure 2B,C, and discussed in detail below) [20,74]. Activation of HR generates single-strand DNA (ssDNA) at multiple steps and requires a specific factor, replication protein A (RPA). In oocytes, the ssDNA binding protein complex RPA is replaced by Rad51 and meiotic cDNA1 (Dmc1). BRCA2 mediates the interaction between Rad51, Dmc1 and ssDNA to form the meiotic presynaptic nucleofilament, resulting in the initiation of HR (Figure 2A). Dmc1 deficiency in mouse oocytes leads to synapsis failure, which is HR-dependent and ultimately reduces follicle survival [75]. 

The role of Rad51 is of paramount importance in the final step of HR and in preventing oocyte death, as is evident from studies in mouse and bovine [48,62,76]. Inhibition of Rad51 prior to irradiation exposure increases damage to DNA, whereas enhancing Rad51 expression by injecting recombinant Rad51 is sufficient to prevent DNA damage [48,76].

In the presence of DSBs, Chk2 activation delays the cell cycle transiently to provide sufficient time for DNA repair [77]. DNA damage checkpoints are primarily expressed when oocytes are in meiotic arrest. Their expression persists at this stage leading to increased sensitivity of oocytes in primordial follicles to DNA damage-inducing agents [75]. Activation of Chk2 simultaneously inhibits cell division cycle (Cdc) phosphatases including Cdc25a, Cdc25b and Cdc25c. This in turn activates cyclin-dependent kinase (Cdk) and consequently blocks the cell cycle progressing from G1 to S and G2/M phase (reviewed in [50]). The activation of p53 family members is another downstream target of ATM and functions to maintain checkpoint activation at G1/S of the cell cycle [53,78]. Inhibition of ATM in mouse oocytes exposed to irradiation results in a failure to activate p63, which then blocks the apoptosis pathway and prevents oocyte death [79]. 

## 4. A Unique p63 Pathway Links DNA Damage and Apoptosis in Oocytes within Primordial Follicles

In conditions resulting in severe DNA damage or with ineffective DNA repair, DNA DSBs accumulation is more likely to initiate the activity of p53 family members. This process is critical to abolish oocytes with unrepaired DNA damage and safeguard against germline mutations. The apoptosis process of oocytes within primordial follicles is mediated by a distinct cell surveillance mechanism involving N-terminal transactivation domain p63 (TAp63α) [24,80,81,82], a p53 family member [83]. TAp63α functions to respond to DNA damage primarily after prophase 1 of meiosis and is constitutively active only in female germ cells once DNA breaks occur [81]. The essential role of TAp63α in the apoptosis process makes it an essential regulator in follicle loss during chemotherapy, which may result in a reduced primordial follicular pool. Oocytes in the quiescent state demonstrate a high TAp63α expression. Wild-type mice exposed to radiation show primordial follicle loss (without loss of growing preantral follicles), whilst TAp63-deficient mice are insensitive to irradiation-induced apoptosis, confirming the indispensable role of TAp63α in the DDR of the oocyte within primordial follicles [80].

The *p63* gene encodes two major isoforms of TAp63, one with the transactivation (TA) domain and the other, ΔN-p63 (N-terminal truncated), lacking the TA domain [84]. TAp63α is the main p63 isoform expressed in the nuclei of oocytes within primordial follicles [80,81,83]. TAp63α is maintained in inactive dimeric form by the transcriptional inhibitory domain (TID) and further stabilised by the interaction of N-terminal transactivation (TAD) with TID and the oligomerization domain. In the dimeric state, the transactivation of TAp63α is suppressed by decreasing its DNA binding affinity and repressing the activity of the domain responsible for the transcriptional process [85]. Exposure to genotoxic agents such as radiation trigger a conformation change in TAp63α to its active tetrameric state, which in turn increases its DNA binding affinity and may ultimately cause apoptosis [85,86,87] and elimination of damaged oocytes (Figure 2C). The presence of TAp63α in oocytes of immature follicles highlights the need for adequate surveillance mechanism to ensure only oocytes with complete DNA damage repair are recruited to ovulation [80,81,84]. 

Mouse oocytes within primordial follicles also express all necessary kinases required to trigger p63 activation. Once DNA damage ensues, it may activate p63 directly, resulting in enhanced oocyte sensitivity to DNA damage compared to granulosa cells [88]. This vulnerability of oocytes to DNA damage is confirmed by a study using a low dose irradiation treatment in mice that is sufficient to induce oocyte death while the surrounding cells of the ovaries are not affected [79]. TAp63α is also expressed in oocytes within primary and preantral follicles, but expression is downregulated with oocyte growth [80,81], resulting in growing oocytes being less sensitive to DNA damage. The sensitivity to DNA damage diminishes once follicles reach the antral stage owing to complete loss of TAp63α expression at this stage [88].

TAp63 activation in oocytes within primordial follicles requires consecutive phosphorylation by Chk2 at serine 582 [89]. TAp63α is not phosphorylated in Chk2 deficient mice following exposure to irradiation [75] with ineffective oocyte elimination, whereas the entire primordial follicle pool in wild type mouse ovary is eradicated [75]. Transcriptional activation of BH3-only pro-apoptotic BCL-2 family members PUMA (p53 upregulated modulator of apoptosis) and NOXA [24] are critical downstream targets of oocytes apoptosis mediated by TAp63 [82]. PUMA and NOXA trigger apoptosis by binding and suppressing the pro-survival B-cell lymphoma 2 (Bcl2) activity, an anti-apoptotic protein implicated in repairing mitochondrial permeability. PUMA and NOXA binding to Bcl-2 unleashes the pro-apoptotic protein B-cell lymphoma (Bcl)-associated X (BAX), precipitating an imbalance between BAX and Bcl2, which then activates apoptosis [90] (Figure 2B). It has been reported that oocytes of PUMA and NOXA deficient mice are not affected by γ-irradiation and are capable of producing healthy offspring [24]. Primordial follicle loss is also much reduced in PUMA knockout mice treated with CP and cisplatin [26]. Alternatively, upregulation of p53 elicits p21 transcription that directly prevents Cdk2 and Cdk4 transcription and eventually induces cycle arrest (reviewed in [50,91]), thus allowing DNA repair [90].

## 5. The PI3K/Akt Pathway Links Primordial Follicle Growth and the DDR

The regulation of recruitment of primordial follicles to grow is strictly controlled by a delicate balance between inhibitory and stimulatory factors to preserve the primordial follicle pool from premature exhaustion. Evidence from genetically modified mice supports the central role of the PTEN/PI3K/Akt signalling pathway in controlling the initiation of primordial follicle growth [93]. Thus, the size of the primordial follicle pool is determined by the dynamic activity of this pathway [17,18]. Accordingly, many studies involving pharmacological and non-pharmacological manipulation of this pathway have been conducted to investigate the activation of primordial follicles in vitro and in vivo [18,31,33,36,94,95,96,97,98,99].

Upregulation of the PI3K/Akt signalling pathway within the oocyte triggers a cascade of reactions that ultimately initiates activation of primordial follicles [6]. PI3K is comprised of a heterodimer of the p85 regulatory subunit and p110 catalytic subunit. In response to growth factors, all regulatory subunits of PI3K interact with the insulin receptor substrate, and thereby activate the catalytic subunit. The interaction induces the phosphorylation of membrane phospholipid phosphatidylinositol 4,5-biphosphonate (PIP2). PIP2 is converted to phosphatidylinositol 3,4,5-trisphosphate (PIP3), which then serves as a second messenger to enable phosphoinositide-dependent kinase 1 (PDK1) activation. PTEN, expressed by the oocyte, reverses this process by converting PIP3 to PIP2. PIP3 binds to Pleckstrin homology (PH) domain of PDK1 and Akt and recruits these two kinases to the subcortical area. This in turn activates PDK1 and subsequent Akt phosphorylation at threonine 308. Akt is further phosphorylated by mammalian target of rapamycin complex 2 (mTORC2) at serine 473 for its full activation, which then regulates a number of downstream targets [6].

PDK1 is indispensable in maintaining primordial follicle survival and preserving reproductive lifespan. It seems likely that both PTEN and PDK1 loss leads to premature ovarian failure (POF) but through different mechanisms. PTEN loss is associated with excessive primordial follicle activation and subsequent follicular atresia, whereas PDK1 deficiency instigates accelerated clearance of primordial follicles straight from their quiescent state [17]. Both types of primordial follicle loss are suggested to underlie ovarian ageing [17]. However, PTEN deletion in oocytes of primary and late stages of growing follicles does not reveal any significant effects on follicular growth [18].

**Figure 3:**
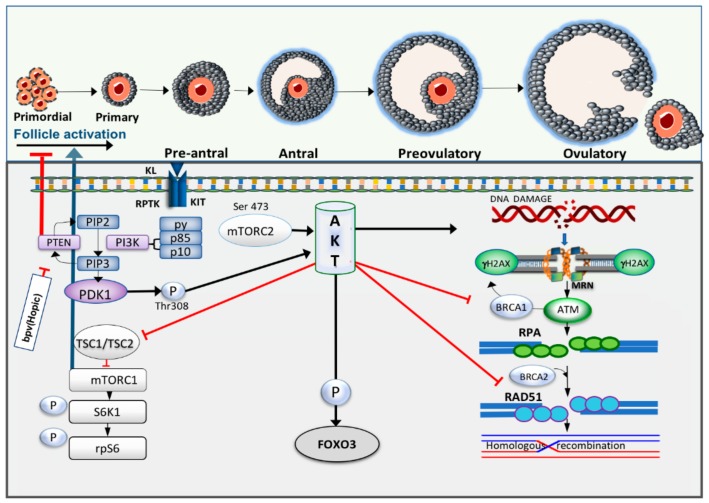
Crosstalk between primordial follicle activation and DDR pathway. Receptor protein tyrosine kinase (RPTK) Kit and its ligand activate phosphoinositide 3-kinase (PI3K) and as a response to this activation, the catalytic subunits of PI3K, p85 and p110, will be activated. In turn, it converts phosphatidylinositol-4,5-bisphosphate (PIP2) to phosphatidylinositol-3,4,5-bisphosphate (PIP3), which then serves as the second messenger to enable phosphoinositide-dependent kinase-1 (PDK1) activation. Phosphatase and tensin homolog deleted on chromosome 10 (PTEN) reverses this process and increases PIP2 expression. PDK1 and Akt are recruited through binding of their pleckstrin homology (PH) domains to PIP3, leading to phosphorylation of protein kinase B (Akt) by PDK1. Akt activation consequently triggers phosphorylation of forkhead box O3 (FOXO3) resulting in cytoplasmic localisation of this transcription factor. Increased in Akt activity also induces phosphorylation of mammalian target of rapamycin complex I (mTORC1) through inactivation of tuberous sclerosis complex 1 and 2 (TSC 1, 2). S6 protein kinase (S6K) activity is then upregulated and simultaneously triggers phosphorylation of ribosomal protein S6 (rpS6). Meanwhile, high intracellular levels of Akt have been reported to increase DNA damage, repress nuclear translocation of breast cancer susceptibility gene 1 (*BRCA1*) and compromise homologous recombination (HR) in breast cancer cells.

mTORC1 is a further downstream substrate of Akt. mTORC1 is upregulated by the destabilisation of the heterodimeric complex of tuberous sclerosis complex 1 (TSC1) and 2 (TSC2). mTORC1 phosphorylates S6 protein kinase (S6K1), which promotes cell growth and proliferation and activates ribosomal protein S6 (rpS6), which increases protein translation [6] (Figure 3). The lack of TSC1 and TSC2 in mouse oocytes instigates massive primordial follicle activation, leading to POF [100]. Forkhead transcription factor FOXO3 (forkhead box O3) is a key target of the PTEN/PI3K/Akt pathway. Once activated, FOXO3 is shuttled from the nucleus to the cytoplasm, which then suppresses its transcriptional function leading to primordial follicle activation [93,101]. The FOXO3 deleted mouse model displays global primordial follicle activation at the neonatal stage leading to primordial follicle loss and POF [102,103]. Conversely, overexpression of constitutively active FOXO3 in the nucleus of mouse oocytes preserves them in a dormant state [104]. FOXO3 can thus be considered as a guardian of the primordial follicle pool, enhancing the ovarian reserve and maintaining reproductive capacity [102,103,104].

PI3K-related protein kinases (PIKKs) are considered to be the main regulators of DNA damage repair capacity of cells. Akt activation implicates the cell cycle checkpoint kinase 1 (Chk1), which has an important role in the DNA damage repair mechanism as it delays the cell cycle progression in S and G2 phase to correct an error of DNA damage before cell division [105]. *PTEN* is a tumour suppressor gene and is an essential factor in promoting normal cell proliferation and coordinating oocyte growth alongside granulosa cell proliferation [18,30]. Oocyte-specific PTEN deletion increases primordial follicle activation and prevents follicles from undergoing apoptosis but may be associated with accelerated clearance of follicles leading to primordial follicle pool exhaustion and POF [17,18]. 

Hyperactivation of Akt due to PTEN inhibition may impair HR activity leading to genomic instability. Notably, a high endogenous level of Akt may be of significant importance in the pathology of cancer as Akt inhibits apoptosis and increases cell proliferation [15]. In cancer cells, excessive Akt activation has also been linked to suppressed NHEJ and DNA DSB repair [106]. PI3K/Akt signalling compromises DNA DSB repair by inactivating the G2 checkpoint [107], with increased Chk1 phosphorylation [108] or cytoplasmic sequestration of BRCA1 [15]. In addition, lack of PTEN in the cell leads to deficient DNA DSBs repair capacity and high incidence of spontaneous DNA breaks [16]. A study using a mouse model has shown that the expression of γH2AX was upregulated by seven-fold in PTEN-null mouse embryonic fibroblasts [109]. Furthermore, PTEN deletion is sufficient to markedly reduce the level of Rad51 that in turn leads to chromosomal instability [110,111]. In normal cells, increased Akt and DNA damage accumulation due to inefficient DNA repair are associated with Ras-induced senescence [112]. Crosstalk between PTEN/PI3K/Akt signalling pathway and DNA damage repair interactions is summarised in Figure 3.

Despite the role of PI3K/Akt in the pathology of cancer, the modulation of this pathway has been adopted as a potential approach for women with premature ovarian insufficiency (POI) and pregnancies have been achieved [34,113]. However, it has become increasingly evident that this pharmacological approach may be detrimental to oocyte/follicle development [29,35,36,37]. We have demonstrated that dipotassium bisperoxo (5-hydroxypyridine-2-carboxyl) oxovanadate (bpv(HOpic)), a potent PTEN inhibitor, compromises the growth of apparently healthy human preantral follicles [36]. Likewise, the use of alginate scaffold and polyethylene glycol (PEG)-fibrinogen to culture human ovarian cortical strips in the presence of 100 μM bpv(HOpic) did not support follicular development [35]. Furthermore, constitutive PI3K activation in the perinatal period in transgenic mouse oocytes leads to lack of co-ordination between oocyte and granulosa cell growth, leading to enlarged oocytes surrounded by immature pre-granulosa cells. These mice are anovulatory, but follicles develop, and oocytes are meiotically competent. The inability to ovulate is likely the result of endocrine factors due to unregulated follicle growth [114]. PI3K over-activation in mouse oocytes has also been associated with granulosa cell tumour (GCT), characterised by excessive granulosa cell proliferation [115].

**Figure 4:**
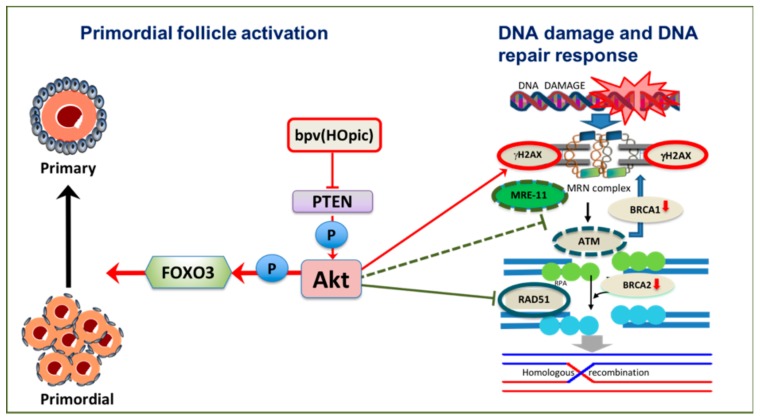
Potential effects of phosphoinositide 3-kinase /protein kinase B (PI3K/Akt) activation on DNA damage and DNA repair response of oocytes in vitro. Inhibition of PTEN by low dose Dipotassium bisperoxo(5-hydroxypyridine-2-carboxyl) oxovanadate (V) (bpv(HOpic)) is sufficient to induce primordial follicle activation. However, gamma H2AX (γH2AX) increases and DNA repair proteins meiotic recombination 11 (MRE11), ataxia telangiectasia mutated (ATM) and Rad51 are downregulated, as are breast cancer susceptibility gene 1 (*BRCA1*) and breast cancer susceptibility gene 2 (*BRCA2*).

A recent finding from our lab utilising bovine ovarian cortical fragments exposed to the PTEN inhibitor bpv(HOpic) for 24 h showed increased primordial follicle activation after six days of culture. However, γH2AX expression in oocytes was upregulated and not associated with increased expression of the DNA repair enzymes ATM and Rad51. A low dose of bpv(HOpic) did not affect BRCA1 and 2 expression and more follicles in this group survived after six days of culture compared to high doses of bpv(HOpic). Nevertheless, a marked decrease in BRCA1 and 2 expression was observed after exposure to high doses suggesting a compromised DDR. Interestingly, despite high γH2AX expression being observed in granulosa cells of secondary stage follicles, DNA repair capacity of these cells was not significantly affected, as indicated by increased MRE11, ATM and Rad51 expression and a non-significant decline of BRCA1 and 2 [29] (Figure 4). Although the mechanism by which PI3K/Akt upregulation induces DNA damage in oocytes has not been elucidated, accelerated primordial follicle growth has been linked to decreased estradiol production indicating impaired granulosa cell function, whilst lowering the activation rate results in normal estradiol production [37]. This suggests that the rapid growth may be associated with a disordered intrafollicular oocyte and somatic cell relationship [116]. This condition may lead to uncoordinated oocyte and granulosa cell growth, as reported in mice [114].

Several publications utilising mouse models have provided evidence that oocytes within resting follicles may be directly targeted by chemotherapy treatments, including CP, cisplatin and doxorubicin [24,26,117,118]. It has also been proposed that primordial follicle depletion following chemotherapy may be induced by the loss of growing follicles with an increase in primordial follicle activation [119,120]. A study investigating the mechanism by which cisplatin induced ovarian failure showed that cisplatin reduced PTEN expression in oocytes leading to primordial follicle activation. Once follicles were activated to grow, they became more vulnerable to apoptosis with a loss of luteinising hormone (LH) receptor expression resulting in decreased oocyte meiotic competence and ovulation failure [23]. These direct and indirect effects of chemotherapy treatments on primordial follicles can form the basis to develop potential methods to protect ovaries against the adverse impacts of chemotherapy [121].

In addition to chemotherapy, another clinical problem that is linked to DNA damage, PI3K/Akt signalling pathway and ovarian ageing is endometriosis. Increased PI3K/Akt activity has been suggested in endometriosis [122,123,124,125,126,127], with loss of nuclear PTEN [128]. Primordial follicle loss in endometriosis has been associated with PI3K/Akt upregulation in mice and human [125] and is suggested to be responsible for ovarian ageing [129]. A diminished ovarian reserve in endometriosis occurs concomitantly with increased DNA damage and compromised DSB repair mechanism, indicated by low Rad51 and BRCA1 expression [130]. Experimental studies in rats indicate that an mTOR inhibitor is effective to suppress the growth of endometriotic implants, supporting the engagement of this pathway [131].

The effects of PI3K/Akt/mTOR on primordial follicle activation following chemotherapy treatment have led to research utilising this mechanism to reduce the adverse impact of chemotherapy on the ovary. In human, as mTOR hyperactivation is a common feature of cancers, mTOR inhibitors are becoming a therapeutic target in certain type of cancers. In a study utilising mouse embryonic fibroblast cell lines, constitutive mTOR activation enhanced apoptosis triggered by chemotherapy through persistent DNA damage as was shown by the upregulation of γH2AX. In parallel, the absence of both PTEN and TSC2 upregulates γH2AX expression. Intriguingly, mTOR inhibition prior to treatment is able to protect cells from etoposide-induced apoptotic cell death [132]. Substrates that inhibit mTOR have been shown to reduce excessive primordial activation and maintain the primordial follicle pool [133,134,135]. This positive effect is due to mTOR downregulation during chemotherapy and subsequently reduced Akt and S6K phosphorylation resulting in decreased primordial follicle loss and maintenance of the ovarian reserve and fertility [136]. Studies investigating the PI3K/Akt pathway are detailed in Table 1.

## 6. DNA Damage Associated with Ovarian Ageing, a Crosstalk between PI3K/Akt/PTEN Signalling, Ageing and DNA Damage Response

Ovarian ageing as a physiological process varies substantially among women depending on the number of primordial follicles and the rate of follicle loss [141,142]. It is also very closely associated with reduced oocyte quality [143]. A link between these is suggested by the increasing rate of primordial follicle activation with age [144] with PI3K/Akt signalling pathway being a key regulator of this growth activation [17,18]. Compromised DNA repair protein interactions as a consequence of ovarian ageing has been connected to increase PI3K/Akt activity [19,20,21,22].

There is increasing evidence of an association between DNA damage and repair capacity of oocytes and maternal age, with DNA repair becoming less efficient with ageing [19,20,21,58]. A study in non-human primates confirmed a lack of DNA repair efficiency with advancing age, with cytoplasmic sequestration of BRCA1 in oocytes [22]. Although DNA damage and repair mechanisms in granulosa cells are not the main focus of this review, it is worth mentioning that γH2AX expression in granulosa cells of growing follicles was not different between old and young mice [22]. This finding may suggest less effective DNA repair in oocytes within primordial follicles compared to surrounding somatic cells. Accordingly, mouse oocytes of all follicle types exhibit high expression of γH2AX with increasing age. At the same time, the oocyte appears to have an ineffective DNA repair mechanism as was shown by a profound drop in BRCA1, MRE11 and ATM but not BRCA2. Mutations in *BRCA1* but not *BRCA2* perturb ovarian stimulation leading to smaller litter size. Interestingly, DNA damage was not evident in pre-granulosa cells within primordial follicles [21]. In line with these findings, the mRNA level of *BRCA1*, *Rad51* and *H2AX* were reduced in aged female rat and buffalo oocytes within primordial follicles [19,58].

Women with *BRCA2* mutations do not show a reduced response to ovarian stimulation [145]. However, BRCA2 deficient mice are able to produce competent and fertilised oocytes but more abnormal embryos are observed [146], indicating an important role of BRCA2 in the oocyte. In women, complete loss of BRCA2 function leads to ovarian dysgenesis resulting in primary amenorrhea, with reduced Rad51 function in HR indicated by low Rad51 expression at the site of DNA damage [147]. A genome-wide association study (GWAS) analysis also shows association between DNA damage repair and age at menopause [148], particularly highlighting links with BRCA1. Likewise, a diminished ovarian reserve mirrored by low AMH levels in women with *BRCA1* but not *BRCA2* mutations [149] supports findings from a transgenic mouse model [21]. In addition, primordial follicles with *BRCA1* mutations are more susceptible to DNA damage accumulation, as shown by high γH2AX expression in primordial follicles [25].

As ageing is thus associated with a reduction in DNA repair capacity, oocytes from older women may be more susceptible to genotoxic insults with increased primordial follicle loss due to apoptosis [10]. It is evident that the degree of doxorubicin induced DNA damage is independent of age, but apoptotic events are more apparent in oocytes of old mice. This may be related to the finding that oocytes from young mice have a greater DNA repair capacity [48]. Qualitative analysis of recent findings of studies in DNA damage and ovarian ageing are summarised in Table 2.

**Figure 5:**
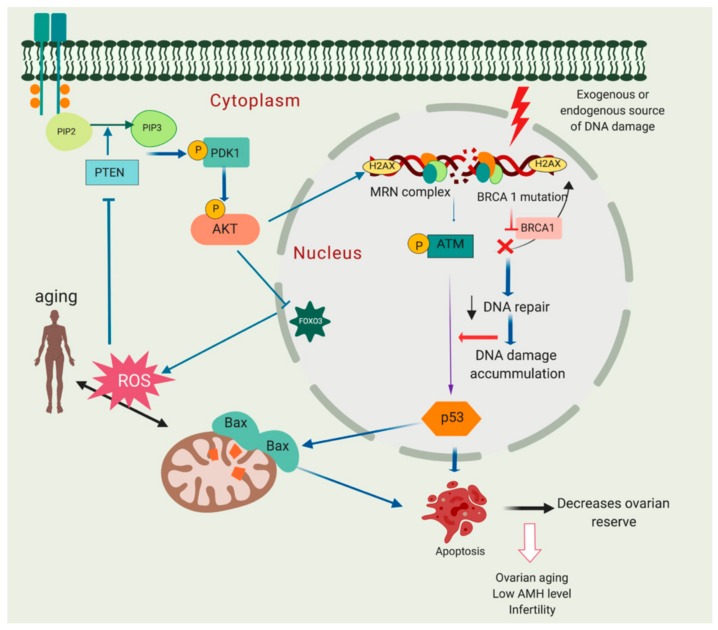
Molecular relationship between phosphoinositide 3-kinase/protein kinase (PTEN/Akt) activation, DNA damage and decreased ovarian reserve. Breast cancer susceptibility gene 1 (*BRCA1*) mutation may lead to compromised DNA repair pathway and eventually primordial follicle apoptosis leading to follicle loss and decreased ovarian reserve. In addition, mitochondria can be one of the major sources of DNA damage. Excessive reactive oxygen species (ROS) production may harm macromolecules in the cells including DNA leading to single-stand breaks (SSBs) or double-strand breaks (DSBs). High ROS expression in mitochondria may lead to PTEN inhibition and increase Akt activation. This may eventually further increase ROS production due to inactivation of forkhead box O3 (FOXO3).

ROS accumulation in mitochondria can be an underlying factor in ageing, by increasing oxidative damage leading to a gradual decrease in follicle quality [150]. Increased ROS activity due to senescence parallels diminished activity of the oxidative defence system and may lead to increased lipid peroxidation, oxidative stress and damage to macromolecules including DNA with either single-strand breaks (SSBs) or DSBs [151]. High ROS expression in follicular fluid of patients undergoing in vitro fertilisation (IVF) has been linked to reduced oocyte fertilisation and poor embryo quality [152]. Mitochondria have been hypothesised to be the first organelle affected by ROS since they are the source of oxygen radical production; ageing is also associated with increased mitochondrial DNA (mtDNA) deletions [153,154]. PTEN upregulation, through modulation of the PI3K/Akt pathway, decreases ROS production in cells (reviewed in [155,156]). Increased ROS concentration in mitochondria due to ageing may inhibit PTEN leading to accumulation of PIP3, which then increases Akt activation and further increases ROS production. This pathway suggests a positive feedback loop between PTEN, PIP3 and ROS [157]. The impact of ageing on the PI3k/Akt signalling pathway, DDR and resting pool depletion is summarised in Figure 5.

## 7. Conclusions and Future Directions

It is clear that mammalian oocytes have distinct DNA damage surveillance mechanisms. There is evidence linking the regulation of primordial follicle growth activation through the PI3K pathway with increased DNA damage/reduced repair, and this provides a model for the development of new approaches to the investigation and potentially therapeutic intervention in both these key aspects of oocyte biology. Evidence from both genetic mouse models and the culture of mammalian ovarian cortical fragments supports the contention that imbalance in signalling events between oocytes and granulosa cells may contribute to impaired follicle function after aberrant primordial follicle growth activation.

Data reviewed here explore the links between the regulation of primordial follicle growth activation and DNA damage repair pathways. The primordial follicle and particularly the oocyte within it has unique physiological challenges, being required to maintain genomic integrity and quality from birth over several decades without cell growth or replication. Thus, the opportunity for repeated DNA surveillance during cell division is absent, and oocyte-specific pathways from DNA damage to apoptosis exist. There is increasing research activity linking follicle growth regulation with oocyte DNA damage and repair capacity in the context of potential prevention of ovarian damage against chemotherapy, radiation or environmental toxicants. The elucidation of the possibility to confer resistance against chemotherapy through identification of key factors in the oocyte apoptotic pathway may lead to clinical trials building on the differences in these pathways between the oocyte and somatic cells.

Rad51, a critical protein involved in oocyte resilience to apoptosis, is a feasible candidate to promote DNA repair capacity in oocytes and ultimately conserve fertility in women undergoing cancer treatment. Administration of recombinant Rad51 into mouse oocytes has been demonstrated to increase DDR, prevent apoptosis, improve the defective DNA repair capacity in oocyte and restore embryo development [48,76]. Future investigation into the safety and efficacy of modulating Rad51 as a clinical application to preserve functional germ cells may be beneficial to improve oocyte and embryo development following chemotherapy exposure and in ageing.

Targeting the PI3K/PTEN/Akt/mTOR pathway, mTOR inhibition with rapamycin [133] and everolimus [136] have also been investigated as a means to protect ovaries during exposure to chemotherapy in mice. Melatonin and ghrelin have also been proposed to protect the ovaries against cisplatin and may also affect this pathway, though perhaps indirectly. Both ghrelin and melatonin suppress cisplatin-mediated PI3K/Akt pathway upregulation and inhibit FOXO3 nuclear shuttling, thus preserving the primordial follicle pool [167]. This is a promising avenue, though it will be essential to ensure that the effects of chemotherapy on cancer cells are not compromised [10].

It has been shown that either complete loss of PUMA or partial loss of TAp63 in mice oocytes could retain the primordial follicle pool following CP and cisplatin exposure. This is a promising approach to reduce the negative effects of chemotherapy on the ovaries [26,117] as the salvage process exclusively occurs within the oocyte without interfering with the cancer treatments [10].

An intriguing novel approach to the protection of ovaries against chemotherapy has been suggested by a recent study introducing microRNAs (miRNAs) [168]. It is reported that miRNAs are differentially expressed in mouse postnatal ovaries exposed to 4-hydroperoxy-cyclophosphamide (4-HC), some of which have been implicated in DDR and apoptosis and affect cellular susceptibility to DNA damaging agents [169]. MiRNAs can be effective techniques as their expression can be adjusted with their microenvironment during chemotherapy treatment, thus minimising off-target toxicity. Lethal 7 (let-7a) mimic is an example of a new miRNA based therapeutic to minimise follicle injury following chemotherapy treatment [170]. However, this work is at an early stage, with challenges including how to deliver miRNAs to a specific target organ with minimum side effects.

In vitro activation (IVA) methods have generated controversy regarding efficacy and safety with in vitro studies indicating that manipulating activation by pharmacological methods has an impact on subsequent quality of oocytes [35,36,37]. Pharmacological primordial activation utilising a PTEN inhibitor has been associated with increased DNA damage and impaired DNA repair capacity particularly in oocytes [29]. While an IVA protocol utilising both PI3K/Akt and Hippo signalling pathways prior to ovarian tissue transplantation may have major negative consequences on follicle health [119,171,172], the PI3K/Akt signalling pathway may also be a potential target to prevent follicle activation and loss following ovarian tissue transplantation, maximising the longevity of the transplanted tissue. A recent study showed that short exposure to a specific inhibitor of mTORC1 partially hindered follicular activation while improving follicle survival and steroidogenesis [37]. Since precocious follicular growth in vitro has been a major constraint in developing in vitro follicle growth systems, lowering the activation rate by using an mTORC inhibitor may have additional value as a promising strategy for the derivation of mature oocytes in vitro. Finally as the canonical PI3K/Akt signalling pathway is interconnected with many feedback loops that are essential for optimal cell function during ageing [157], future research investigating the potential of manipulation of PTEN and PI3K to reduce ROS accumulation and thus damage in ageing oocytes will be essential.

## Figures and Tables

**Table 1:** Recent studies investigating the impact of phosphatase and tensin homolog deleted on chromosome 10/phosphoinositide 3-kinase/protein kinase B/mammalian target of rapamycin complex (PTEN/PI3K/Akt/mTORC) pathway either as a part of genetic modification/pharmacological activation, chemotherapy treatment or ovotoxicity exposure on primordial follicle activation, follicular growth and survival.

Agents Used/Compounds/Concentration	Mechanism of Action	Species/Methods	Effects on Follicular Growth/Survival	Specific Effects on Granulosa Cells/Oocyte	Study
1 and 10 μM Dipotassium bisperoxo (5-hydroxypyridine-2-carboxyl) oxovanadate (V) (bpv(HOpic)) for 24 h/ PTEN inhibitors.	Increase PI3K/Akt	Bovine/ovarian cortical fragments cultured.	Decreases in higher dose.	Compromises DNA damage response (DDR).	[29]
20, 40, 60, 80, 120 and 140 μM diazinon (DZN)	Inhibit PI3K/Akt	Porcine isolated granulosa cells.	Granulosa cells death	Increase DNA damage, mRNA level of Ataxia telangiectasia mutated (ATM), Rad51 and breast cancer susceptibility gene1 (*BRCA1*) increase p53 leading to granulosa cell death.	[137]
30 μM bpv (HOpic) + 150 μg/mL 740Y-P for 24 and 48 h or 100 nM everolimus.	Increase PI3K/Akt and inhibit mTOR activation respectively	Cryopreserved human ovarian cortical fragments cultured	Lowering the rate of activation improves follicular growth.	PTEN inhibition compromises granulosa cell estradiol production.	[37]
Cyclophosphamide (CP) 75 mg, 100 mg, 150 mg per kg body weight and 5 mg/kg body weight per day 1 week before and after CP administration.	PI3K/Akt activation	Mice, in vivo	CP induces non-growing and growing follicle loss. Rapamycin prevents CP induced primordial follicle activation.	Anti-mullerian hormone (AMH) expression decreases after CP exposure.	[133]
Transgenic mouse model	Increase PI3K activation in transgenic mice, Cre+	Transgenic mice, Cre+ and Cre−	Normal secondary follicles, granulosa cell tumour (GCT) in primordial and primary follicles.	Bilateral GCT due to increased activin A.	[115]
440 μM bisphenol A(BPA).	Increases PI3K/Akt activation	Rat ovarian fragment culture exposed to BPA.	BPA induces DNA damage both in oocytes and granulosa cells. PI3K signalling pathway involved in BPA-induced DNA damage.	Primordial follicle is activated to replace the larger follicle depletion.	[138]
Transgenic mouse model	Increase PI3K activation in transgenic mice, Cre+	Transgenic mice, Cre+ and Cre−	Increases follicles survival	Asynchronous oocytes and granulosa cells growth.	[114]
100 μM bpv(HOpic) for 25 h	Increase PI3K/Akt activation	Human ovarian cortical fragments cultured.	No damage to the follicular growth.	Enhance estradiol production without any damage to follicles compared to control group.	[33]
200 μM phosphatidic acid (PA) and 50 μM propranolol (PRO) for 24 h in mice; bpv(HOpic)(100 μM) /740Y-P (250 μM /mL) for 24 h, 740Y-P (250 μM /mL) only for another 24 h; PA (100 mM)/740Y-P (200 μM)/PRO (50 μM) for 24 h in human.	Increase PI3K/Akt /mTOR activation.	Mice and human ovaries transiently incubated in mTOR activators followed by grafting into female mice.	No damage to the follicular growth.	NA	[139]
30 μM of bpv(HOpic), and 150 μM /mL of 740YP for 24 h followed by incubation with 740YP alone for another 24 h	Increase PI3K/Akt activation.	Human ovarian cortical fragments transplantation following in vitro activation (IVA).	Autografting of ovarian fragments following in vitro activation (IVA) procedure to infertility related primary ovarian insufficiency (POI) patients.	NA	[34]
1 μM bpv(HOpic) and 10 and 100 μM bpv(HOpic) (unpublished)	Increase PI3K/Akt activation	Human ovarian cortical fragments and isolated preantral follicle culture.	Higher dose compromises follicular growth. The lower dose is associated with deleterious effects on subsequent growth of preantral follicles.	NA	[36]
Cisplatin, once daily at doses of 0.5, 1.0, 1.5 and 2.0 mg/ kg for 5 to 14 days	Activation of PI3K/Akt	Intraperitoneal injection of cisplatin in mice	Increases the proportion of growing follicles.	Induces ovarian failure.	[23]
100 μM bpv(HOpic) and 500 μM /mL 740Y-P for 24 and 48 h	Increase PI3K/Akt activation.	Human ovarian cortical fragments cultured in polyethylene glycol (PEG)-fibrinogen hydrogels.	Compromises follicle survival.	NA	[35]
30 μM bpv(HOpic) and 150 μg/mL 740YP for 24 h	Increase PI3K/Akt activation.	Mice ovarian transplantation and human ovarian fragments transplantation following IVA.	Promotes primordial follicle activation both in mice and human.	NA	[113]
Female mice deficient in PTEN	Increase PI3K/Akt activation	PTEN knockout mice	Rapamycin reduces the primordial follicles activation in PTEN knockout mice.	Rapamycin prevents global primordial follicles activation induced by the absence of PTEN.	[134]
1 μM bpv(HOpic) for 24 h	Increase PI3K/Akt activity.	Mice cortical fragments IVA followed by transplantation and bpv(HOpic) directly injected to female mice.	Does not compromise follicular health.	More mature and fertilised oocytes in PTEN inhibition group.	[32]
100 μM bpv(HOpic) and/or 500 μg/mL 740Y-P for 48 h or bpv(HOpic) plus 740Y-P together with the Akt inhibitor SH-550 μM or the PI3K inhibitor Wortmannin 25 μM.	Increase PI3K/Akt, Akt inhibitor decreases the activation.	Mice and human cortical fragments incubated in Akt activators followed by xenografting.	Increases in the number of secondary and antral stage follicles following xenografting and does not affect follicular health.	No malignancy observed after long term ovarian transplantation.	[140]
Mice lacking Tuberous sclerosis complex 1 (TSC1), PTEN; TSC1 and PTEN; Phosphatidylinositol-dependent kinase 1 (PDK1) and PDK1 and TSC1 in oocytes.	Enhances mTOR activation.	Mutant female mice	Degenerated activated primordial follicles (short term), diminished follicular health (long term).	Rapamycin prevents global primordial follicle activation. Activation does not cause tumour development.	[100]
Homozygous mutant female mice deficient Tuberous sclerosis complex 2 ( TSC2) in oocytes.	Enhances mTOR activation.	Mutant female mice.	Massive primordial follicle activation.	Depletion of follicle reserve.	[93]
Female mice lacking PTEN, PDK1 and r ibosomal protein S6 kinase (rpS6)	Increases PI3K/Akt	Mutant female mice.	Follicles with degenerating oocytes in PDK1 deletion and enlarged oocytes in PTEN deletion.	The absence of PTEN causes Primary Ovarian Insufficiency (POI) that can be reversed by PDK1 deletion.	[17]
PTEN deletion in mice.	Increases PI3K/Akt	PTEN mutant mice	Tends to be normal follicle morphology but with enlarged oocytes and flattened granulosa cells.	PTEN deletion leads to excessive primordial follicle activation.	[18]

NA: Not available.

**Table 2:** Summary of recent clinical and experimental studies providing evidence linking DNA damage response (DDR), ovarian ageing and ovarian reserve.

Study Focus	Study Type	DDR Pathway Affected	Main Outcomes	References
Oocyte maturation rate of breast cancer patient with breast cancer susceptibility gene 1 (*BRCA1*) and Breast cancer 2 (*BRCA2*) mutation.	Retrospective cohort study.	BRCA1 and BRCA2	The number of mature oocytes resulted from in vitro maturation (IVM) procedure is not different between women with *BRCA1* and without *BRCA* mutation.	[158]
Ovarian reserve of patients with *BRCA* mutation carriers and non-carriers with or without malignancy.	Retrospective cohort study.	BRCA1 and BRCA2	Patients with *BRCA* mutation carriers and noncarriers show comparable ovarian reserve and number of oocytes yield following ovarian stimulation.	[159]
The role of BRCA2 in ovarian development and puberty onset.	A case control study in human.	BRCA2 and Rad51	Lack of BRCA2 reduces Rad51 recruitment during homologous recombination.	[147]
Ovarian reserve in patients with *BRCA1* mutation.	Case-control study.	γH2AX, BRCA1 and BRCA2	DNA double-strand breaks (DSBs) increase in *BRCA1* mutation group but not *BRCA2*. DNA damage increases with age in *BRCA1/2* mutation.	[25]
Oocyte yield following ovarian stimulation in patients with *BRCA1/2* mutation.	Retrospective cohort study.	BRCA1 and BRCA2	The number of oocytes produced by women with *BRCA* mutation is lower than without BRCA1 mutation.	[160]
DNA damage and repair capacity of aged and young buffalo ovaries.	Experimental study in buffalo ovaries.	BRCA1, γH2AX, MRE11, Rad51 and ATM	mRNA expression of BRCA1, meiotic recombination 11 (MRE11), Rad51 and ataxia telangiectasia mutated (ATM) decline significantly in aged buffalo ovaries.	[58]
The effects of *BRCA* mutation on anti-Mullerian hormone (AMH) serum level.	Prospective cohort study	BRCA1 and BRCA2	Patients with *BRCA2* mutations exhibit a lower AMH level compare to low-risk control patients.	[161]
Anti-mullerian hormone (AMH) serum level in patients with *BRCA1/2* mutation.	Cross-sectional study	BRCA1 and BRCA2	AMH serum level of patients with *BRCA1/2* mutation carriers does not significantly different from non-carriers.	[162]
AMH serum level in women with *BRCA1* and *BRCA2* mutation.	Cross-sectional study	BRCA1 and BRCA2	*BRCA1* but not in *BRCA2* mutation carriers have a lower AMH level.	[149]
Ovarian ageing effects on DNA damage repair response in rat ovaries.	Experimental	γH2AX, BRCA1, MRE11, Rad51, ATM, BRCA1 and BRCA2	DNA repair proteins BRCA1, Rad51, ATM and γH2AX in aged rat primordial follicles declined compared to immature rats.	[19]
Comparison of proteins profile of primordial follicles isolated from immature rat and aged rat.	Experimental	Heat shock cognate 71kDa (Hsp71C), calreticulin, Bcl-2-related ovarian killer protein (BOK)	Protein expression for DSBs response decreases significantly in aged rats.	[59]
The association between DNA DSBs in granulosa cells and ageing.	Experimental	γH2AX, BRCA1, Telomeric repeat binding factor (TRF2)	Increased γH2AX and decreased BRCA1 expression in all follicle types with age.	[22]
The association between AMH serum level and *BRCA* mutation.	Cross-sectional study	BRCA1 and BRCA2	AMH serum level of patients with a *BRCA1* mutation is lower than without *BRCA1* mutation.	[163]
The effect of ovarian ageing on DNA DSBs of oocytes and granulosa cells.	Experimental	γH2AX, BRCA1, MRE11, Rad51, ATM, BRCA1 and BRCA2	Increased DNA damage and decreased DDR capacity with advancing age.	[21]
Time to menopause in *BRCA1* and 2 mutations carriers.	Case control study.	BRCA1 and BRCA2	Both *BRCA1* and *2* mutation patients experience menopause earlier than control.	[164]
Doxorubicin effects on ovarian ageing.	Experimental	γH2AX, ATM and activated caspase 3	γH2AX expression is higher in ovarian tissue exposed to doxorubicin in vitro.	[118]
Transactivation p73 (TAp73) expression in young and aged female oocytes.	Experimental	TAp73	TAp73 is downregulated in older women’s oocytes.	[165]
The effects of age on the occurrence of aneuploidy in mouse oocytes.	Experimental	BRCA1	*BRCA1* expression is decreased in oocytes of aged mice. Aneuploidy increases in aged oocytes.	[166]
The role of BRCA2 in male and female gametogenesis.	Experimental	BRCA2	BRCA2 deficiency in mice leads to infertility.	[146]

## References

[1] Telfer E.E., Zelinski M.B. (2013). Ovarian follicle culture: Advances and challenges for human and nonhuman primates. Fertil. Steril..

[2] Faddy M.J., Gosden R.G. (1995). A mathematical model of follicle dynamics in the human ovary. Hum. Reprod..

[3] Hansen K.R., Knowlton N.S., Thyer A.C., Charleston J.S., Soules M.R., Klein N.A. (2008). A new model of reproductive aging: The decline in ovarian non-growing follicle number from birth to menopause. Hum. Reprod..

[4] Broekmans F.J., Knauff E.A., te Velde E.R., Macklon N.S., Fauser B.C. (2007). Female reproductive ageing: Current knowledge and future trends. Trends Endocrinol. Metab..

[5] McGee E.A., Hsueh A.J. (2000). Initial and cyclic recruitment of ovarian follicles. Endocr. Rev..

[6] Adhikari D., Liu K. (2009). Molecular mechanisms underlying the activation of mammalian primordial follicles. Endocr. Rev..

[7] Wang Q., Sun Q.Y. (2007). Evaluation of oocyte quality: Morphological, cellular and molecular predictors. Reprod. Fertil. Dev..

[8] Ashwood-Smith M.J., Edwards R.G. (1996). DNA repair by oocytes. Mol. Hum. Reprod..

[9] Tilly J.L. (2001). Commuting the death sentence: How oocytes strive to survive. Nat. Rev. Mol. Cell Biol..

[10] Winship A.L., Stringer J.M., Liew S.H., Hutt K.J. (2018). The importance of DNA repair for maintaining oocyte quality in response to anti-cancer treatments, environmental toxins and maternal ageing. Hum. Reprod. Update.

[11] Dupont J., Scaramuzzi R.J. (2016). Insulin signalling and glucose transport in the ovary and ovarian function during the ovarian cycle. Biochem. J..

[12] Stokoe D. (2005). The phosphoinositide 3-kinase pathway and cancer. Expert. Rev. Mol. Med..

[13] Engelman J.A., Luo J., Cantley L.C. (2006). The evolution of phosphatidylinositol 3-kinases as regulators of growth and metabolism. Nat. Rev. Genet..

[14] Karimian A., Mir S.M., Parsian H., Refieyan S., Mirza-Aghazadeh-Attari M., Yousefi B., Majidinia M. (2019). Crosstalk between phosphoinositide 3-kinase/akt signaling pathway with DNA damage response and oxidative stress in cancer. J. Cell. Biochem..

[15] Plo I., Laulier C., Gauthier L., Lebrun F., Calvo F., Lopez B.S. (2008). Akt1 inhibits homologous recombination by inducing cytoplasmic retention of brca1 and rad51. Cancer Res..

[16] Puc J., Keniry M., Li H.S., Pandita T.K., Choudhury A.D., Memeo L., Mansukhani M., Murty V.V., Gaciong Z., Meek S.E. (2005). Lack of pten sequesters chk1 and initiates genetic instability. Cancer Cell..

[17] Reddy P., Adhikari D., Zheng W., Liang S., Hamalainen T., Tohonen V., Ogawa W., Noda T., Volarevic S., Huhtaniemi I. (2009). Pdk1 signaling in oocytes controls reproductive aging and lifespan by manipulating the survival of primordial follicles. Hum. Mol. Genet..

[18] Reddy P., Liu L., Adhikari D., Jagarlamudi K., Rajareddy S., Shen Y., Du C., Tang W., Hamalainen T., Peng S.L. (2008). Oocyte-specific deletion of pten causes premature activation of the primordial follicle pool. Science.

[19] Govindaraj V., Keralapura Basavaraju R., Rao A.J. (2015). Changes in the expression of DNA double strand break repair genes in primordial follicles from immature and aged rats. Reprod. Biomed. Online.

[20] Oktay K., Turan V., Titus S., Stobezki R., Liu L. (2015). Brca mutations, DNA repair deficiency, and ovarian aging. Biol. Reprod..

[21] Titus S., Li F., Stobezki R., Akula K., Unsal E., Jeong K., Dickler M., Robson M., Moy F., Goswami S. (2013). Impairment of brca1-related DNA double-strand break repair leads to ovarian aging in mice and humans. Sci. Transl. Med..

[22] Zhang D., Zhang X., Zeng M., Yuan J., Liu M., Yin Y., Wu X., Keefe D.L., Liu L. (2015). Increased DNA damage and repair deficiency in granulosa cells are associated with ovarian aging in rhesus monkey. J. Assist. Reprod. Genet..

[23] Chang E.M., Lim E., Yoon S., Jeong K., Bae S., Lee D.R., Yoon T.K., Choi Y., Lee W.S. (2015). Cisplatin induces overactivation of the dormant primordial follicle through pten/akt/foxo3a pathway which leads to loss of ovarian reserve in mice. PLoS ONE.

[24] Kerr J.B., Hutt K.J., Michalak E.M., Cook M., Vandenberg C.J., Liew S.H., Bouillet P., Mills A., Scott C.L., Findlay J.K. (2012). DNA damage-induced primordial follicle oocyte apoptosis and loss of fertility require tap63-mediated induction of puma and noxa. Mol. Cell..

[25] Lin W., Titus S., Moy F., Ginsburg E.S., Oktay K. (2017). Ovarian aging in women with brca germline mutations. J. Clin. Endocrinol. Metab..

[26] Nguyen Q.N., Zerafa N., Liew S.H., Morgan F.H., Strasser A., Scott C.L., Findlay J.K., Hickey M., Hutt K.J. (2018). Loss of puma protects the ovarian reserve during DNA-damaging chemotherapy and preserves fertility. Cell Death Dis..

[27] Rinaldi V.D., Hsieh K., Munroe R., Bolcun-Filas E., Schimenti J.C. (2017). Pharmacological inhibition of the DNA damage checkpoint prevents radiation-induced oocyte death. Genetics.

[28] Wang Y., Liu M., Johnson S.B., Yuan G., Arriba A.K., Zubizarreta M.E., Chatterjee S., Nagarkatti M., Nagarkatti P., Xiao S. (2019). Doxorubicin obliterates mouse ovarian reserve through both primordial follicle atresia and overactivation. Toxicol. Appl. Pharmacol..

[29] Maidarti M., Clarkson Y.L., McLaughlin M., Anderson R.A., Telfer E.E. (2019). Inhibition of pten activates bovine non-growing follicles in vitro but increases DNA damage and reduces DNA repair response. Hum. Reprod..

[30] Jagarlamudi K., Liu L., Adhikari D., Reddy P., Idahl A., Ottander U., Lundin E., Liu K. (2009). Oocyte-specific deletion of pten in mice reveals a stage-specific function of pten/pi3k signaling in oocytes in controlling follicular activation. PLoS ONE.

[31] Jagarlamudi K., Reddy P., Adhikari D., Liu K. (2010). Genetically modified mouse models for premature ovarian failure (pof). Mol. Cell. Endocrinol..

[32] Adhikari D., Gorre N., Risal S., Zhao Z., Zhang H., Shen Y., Liu K. (2012). The safe use of a pten inhibitor for the activation of dormant mouse primordial follicles and generation of fertilizable eggs. PLoS ONE.

[33] Novella-Maestre E., Herraiz S., Rodriguez-Iglesias B., Diaz-Garcia C., Pellicer A. (2015). Short-term pten inhibition improves in vitro activation of primordial follicles, preserves follicular viability, and restores amh levels in cryopreserved ovarian tissue from cancer patients. PLoS ONE.

[34] Suzuki N., Yoshioka N., Takae S., Sugishita Y., Tamura M., Hashimoto S., Morimoto Y., Kawamura K. (2015). Successful fertility preservation following ovarian tissue vitrification in patients with primary ovarian insufficiency. Hum. Reprod..

[35] Lerer-Serfaty G., Samara N., Fisch B., Shachar M., Kossover O., Seliktar D., Ben-Haroush A., Abir R. (2013). Attempted application of bioengineered/biosynthetic supporting matrices with phosphatidylinositol-trisphosphate-enhancing substances to organ culture of human primordial follicles. J. Assist. Reprod. Genet..

[36] McLaughlin M., Kinnell H.L., Anderson R.A., Telfer E.E. (2014). Inhibition of phosphatase and tensin homologue (pten) in human ovary in vitro results in increased activation of primordial follicles but compromises development of growing follicles. Mol. Hum. Reprod..

[37] Grosbois J., Demeestere I. (2018). Dynamics of pi3k and hippo signaling pathways during in vitro human follicle activation. Hum. Reprod..

[38] Brandmaier A., Hou S.Q., Shen W.H. (2017). Cell cycle control by pten. J. Mol. Biol..

[39] Yin Y., Shen W.H. (2008). Pten: A new guardian of the genome. Oncogene.

[40] Adriaens I., Smitz J., Jacquet P. (2009). The current knowledge on radiosensitivity of ovarian follicle development stages. Hum. Reprod. Update.

[41] Jackson S.P., Bartek J. (2009). The DNA-damage response in human biology and disease. Nature.

[42] Khanna K.K., Jackson S.P. (2001). DNA double-strand breaks: Signaling, repair and the cancer connection. Nat. Genet..

[43] Menezo Y., Dale B., Cohen M. (2010). DNA damage and repair in human oocytes and embryos: A review. Zygote.

[44] Moher D., Shamseer L., Clarke M., Ghersi D., Liberati A., Petticrew M., Shekelle P., Stewart L.A., Group P.-P. (2015). Preferred reporting items for systematic review and meta-analysis protocols (prisma-p) 2015 statement. Syst. Rev..

[45] Branzei D., Foiani M. (2008). Regulation of DNA repair throughout the cell cycle. Nat. Rev. Mol. Cell. Biol..

[46] Lindahl T., Barnes D.E. (2000). Repair of endogenous DNA damage. Cold Spring Harb. Symp. Quant. Biol..

[47] Oktay K., Moy F., Titus S., Stobezki R., Turan V., Dickler M., Goswami S. (2014). Age-related decline in DNA repair function explains diminished ovarian reserve, earlier menopause, and possible oocyte vulnerability to chemotherapy in women with brca mutations. J. Clin. Oncol..

[48] Kujjo L.L., Laine T., Pereira R.J., Kagawa W., Kurumizaka H., Yokoyama S., Perez G.I. (2010). Enhancing survival of mouse oocytes following chemotherapy or aging by targeting bax and rad51. PLoS ONE.

[49] Martin J.H., Bromfield E.G., Aitken R.J., Lord T., Nixon B. (2018). Double strand break DNA repair occurs via non-homologous end-joining in mouse mii oocytes. Sci. Rep..

[50] Collins J.K., Jones K.T. (2016). DNA damage responses in mammalian oocytes. Reproduction.

[51] Lieber M.R. (2010). The mechanism of double-strand DNA break repair by the nonhomologous DNA end joining pathway. Annu. Rev. Biochem..

[52] Rodgers K., McVey M. (2016). Error-prone repair of DNA double-strand breaks. J. Cell. Physiol..

[53] Heijink A.M., Krajewska M., van Vugt M.A. (2013). The DNA damage response during mitosis. Mutat. Res..

[54] Bekker-Jensen S., Mailand N. (2010). Assembly and function of DNA double-strand break repair foci in mammalian cells. DNA Repair.

[55] Stringer J.M., Winship A., Liew S.H., Hutt K. (2018). The capacity of oocytes for DNA repair. Cell. Mol. Life Sci..

[56] Menezo Y., Russo G., Tosti E., El Mouatassim S., Benkhalifa M. (2007). Expression profile of genes coding for DNA repair in human oocytes using pangenomic microarrays, with a special focus on ros linked decays. J. Assist. Reprod. Genet..

[57] Jaroudi S., Kakourou G., Cawood S., Doshi A., Ranieri D.M., Serhal P., Harper J.C., SenGupta S.B. (2009). Expression profiling of DNA repair genes in human oocytes and blastocysts using microarrays. Hum. Reprod..

[58] Govindaraj V., Krishnagiri H., Chauhan M.S., Rao A.J. (2017). Brca-1 gene expression and comparative proteomic profile of primordial follicles from young and adult buffalo (bubalus bubalis) ovaries. Anim. Biotechnol..

[59] Govindaraj V., Rao A.J. (2015). Comparative proteomic analysis of primordial follicles from ovaries of immature and aged rats. Syst. Biol. Reprod. Med..

[60] Fiorenza M.T., Bevilacqua A., Bevilacqua S., Mangia F. (2001). Growing dictyate oocytes, but not early preimplantation embryos, of the mouse display high levels of DNA homologous recombination by single-strand annealing and lack DNA nonhomologous end joining. Dev. Biol..

[61] Rimon-Dahari N., Yerushalmi-Heinemann L., Alyagor L., Dekel N. (2016). Ovarian folliculogenesis. Results Probl. Cell. Differ..

[62] Perez G.I., Acton B.M., Jurisicova A., Perkins G.A., White A., Brown J., Trbovich A.M., Kim M.R., Fissore R., Xu J. (2007). Genetic variance modifies apoptosis susceptibility in mature oocytes via alterations in DNA repair capacity and mitochondrial ultrastructure. Cell. Death Differ..

[63] Turan V., Oktay K. (2019). Brca-related atm-mediated DNA double-strand break repair and ovarian aging. Hum. Reprod. Update.

[64] Goedecke W., Vielmetter W., Pfeiffer P. (1992). Activation of a system for the joining of nonhomologous DNA ends during xenopus egg maturation. Mol. Cell. Biol..

[65] So S., Davis A.J., Chen D.J. (2009). Autophosphorylation at serine 1981 stabilizes atm at DNA damage sites. J. Cell Biol..

[66] You Z., Chahwan C., Bailis J., Hunter T., Russell P. (2005). Atm activation and its recruitment to damaged DNA require binding to the c terminus of nbs1. Mol. Cell Biol..

[67] Inagaki A., Roset R., Petrini J.H. (2016). Functions of the mre11 complex in the development and maintenance of oocytes. Chromosoma.

[68] Ganesan S., Keating A.F. (2016). The ovarian DNA damage repair response is induced prior to phosphoramide mustard-induced follicle depletion, and ataxia telangiectasia mutated inhibition prevents pm-induced follicle depletion. Toxicol. Appl. Pharmacol..

[69] Rogakou E.P., Pilch D.R., Orr A.H., Ivanova V.S., Bonner W.M. (1998). DNA double-stranded breaks induce histone h2ax phosphorylation on serine 139. J. Biol. Chem..

[70] Tomilin N.V., Solovjeva L.V., Svetlova M.P., Pleskach N.M., Zalenskaya I.A., Yau P.M., Bradbury E.M. (2001). Visualization of focal nuclear sites of DNA repair synthesis induced by bleomycin in human cells. Radiat. Res..

[71] Nazarov I.B., Smirnova A.N., Krutilina R.I., Svetlova M.P., Solovjeva L.V., Nikiforov A.A., Oei S.L., Zalenskaya I.A., Yau P.M., Bradbury E.M. (2003). Dephosphorylation of histone gamma-h2ax during repair of DNA double-strand breaks in mammalian cells and its inhibition by calyculin a. Radiat. Res..

[72] Jungmichel S., Stucki M. (2010). Mdc1: The art of keeping things in focus. Chromosoma.

[73] Marangos P., Carroll J. (2012). Oocytes progress beyond prophase in the presence of DNA damage. Curr. Biol..

[74] Jazayeri A., Falck J., Lukas C., Bartek J., Smith G.C., Lukas J., Jackson S.P. (2006). Atm- and cell cycle-dependent regulation of atr in response to DNA double-strand breaks. Nat. Cell Biol..

[75] Bolcun-Filas E., Rinaldi V.D., White M.E., Schimenti J.C. (2014). Reversal of female infertility by chk2 ablation reveals the oocyte DNA damage checkpoint pathway. Science.

[76] Kujjo L.L., Ronningen R., Ross P., Pereira R.J., Rodriguez R., Beyhan Z., Goissis M.D., Baumann T., Kagawa W., Camsari C. (2012). Rad51 plays a crucial role in halting cell death program induced by ionizing radiation in bovine oocytes. Biol. Reprod..

[77] Zannini L., Delia D., Buscemi G. (2014). Chk2 kinase in the DNA damage response and beyond. J. Mol. Cell Biol..

[78] Basu A., Haldar S. (1998). The relationship between bci2, bax and p53: Consequences for cell cycle progression and cell death. Mol. Hum. Reprod..

[79] Kim D.A., Suh E.K. (2014). Defying DNA double-strand break-induced death during prophase i meiosis by temporal tap63alpha phosphorylation regulation in developing mouse oocytes. Mol. Cell. Biol..

[80] Suh E.K., Yang A., Kettenbach A., Bamberger C., Michaelis A.H., Zhu Z., Elvin J.A., Bronson R.T., Crum C.P., McKeon F. (2006). P63 protects the female germ line during meiotic arrest. Nature.

[81] Livera G., Petre-Lazar B., Guerquin M.J., Trautmann E., Coffigny H., Habert R. (2008). P63 null mutation protects mouse oocytes from radio-induced apoptosis. Reproduction.

[82] Amelio I., Grespi F., Annicchiarico-Petruzzelli M., Melino G. (2012). P63 the guardian of human reproduction. Cell Cycle.

[83] Levine A.J., Tomasini R., McKeon F.D., Mak T.W., Melino G. (2011). The p53 family: Guardians of maternal reproduction. Nat. Rev. Mol. Cell Biol..

[84] Gebel J., Tuppi M., Krauskopf K., Coutandin D., Pitzius S., Kehrloesser S., Osterburg C., Dotsch V. Control mechanisms in germ cells mediated by p53 family proteins. J. Cell Sci..

[85] Deutsch G.B., Zielonka E.M., Coutandin D., Dotsch V. (2011). Quality control in oocytes: Domain-domain interactions regulate the activity of p63. Cell Cycle.

[86] Straub W.E., Weber T.A., Schafer B., Candi E., Durst F., Ou H.D., Rajalingam K., Melino G., Dotsch V. (2010). The c-terminus of p63 contains multiple regulatory elements with different functions. Cell Death Dis..

[87] Deutsch G.B., Zielonka E.M., Coutandin D., Weber T.A., Schafer B., Hannewald J., Luh L.M., Durst F.G., Ibrahim M., Hoffmann J. (2011). DNA damage in oocytes induces a switch of the quality control factor tap63alpha from dimer to tetramer. Cell.

[88] Coutandin D., Osterburg C., Srivastav R.K., Sumyk M., Kehrloesser S., Gebel J., Tuppi M., Hannewald J., Schafer B., Salah E. (2016). Quality control in oocytes by p63 is based on a spring-loaded activation mechanism on the molecular and cellular level. Elife.

[89] Tuppi M., Kehrloesser S., Coutandin D.W., Rossi V., Luh L.M., Strubel A., Hotte K., Hoffmeister M., Schafer B., De Oliveira T. (2018). Oocyte DNA damage quality control requires consecutive interplay of chk2 and ck1 to activate p63. Nat. Struct. Mol. Biol..

[90] Nowsheen S., Yang E. (2012). The intersection between DNA damage response and cell death pathways. Exp. Oncol..

[91] Guo X., Keyes W.M., Papazoglu C., Zuber J., Li W., Lowe S.W., Vogel H., Mills A.A. (2009). Tap63 induces senescence and suppresses tumorigenesis in vivo. Nat. Cell Biol..

[92] Tavana O., Benjamin C.L., Puebla-Osorio N., Sang M., Ullrich S.E., Ananthaswamy H.N., Zhu C. (2010). Absence of p53-dependent apoptosis leads to uv radiation hypersensitivity, enhanced immunosuppression and cellular senescence. Cell Cycle.

[93] Adhikari D., Flohr G., Gorre N., Shen Y., Yang H., Lundin E., Lan Z., Gambello M.J., Liu K. (2009). Disruption of tsc2 in oocytes leads to overactivation of the entire pool of primordial follicles. Mol. Hum. Reprod..

[94] Dole G., Nilsson E.E., Skinner M.K. (2008). Glial-derived neurotrophic factor promotes ovarian primordial follicle development and cell-cell interactions during folliculogenesis. Reproduction.

[95] Nilsson E., Parrott J.A., Skinner M.K. (2001). Basic fibroblast growth factor induces primordial follicle development and initiates folliculogenesis. Mol. Cell. Endocrinol..

[96] Ojeda S.R., Romero C., Tapia V., Dissen G.A. (2000). Neurotrophic and cell-cell dependent control of early follicular development. Mol. Cell. Endocrinol..

[97] Nilsson E.E., Skinner M.K. (2004). Kit ligand and basic fibroblast growth factor interactions in the induction of ovarian primordial to primary follicle transition. Mol. Cell. Endocrinol..

[98] Nilsson E.E., Kezele P., Skinner M.K. (2002). Leukemia inhibitory factor (lif) promotes the primordial to primary follicle transition in rat ovaries. Mol. Cell. Endocrinol..

[99] McLaughlin E.A., McIver S.C. (2009). Awakening the oocyte: Controlling primordial follicle development. Reproduction.

[100] Adhikari D., Zheng W., Shen Y., Gorre N., Hamalainen T., Cooney A.J., Huhtaniemi I., Lan Z.J., Liu K. (2010). Tsc/mtorc1 signaling in oocytes governs the quiescence and activation of primordial follicles. Hum. Mol. Genet..

[101] Adhikari D., Liu K. (2010). Mtor signaling in the control of activation of primordial follicles. Cell Cycle.

[102] Castrillon D.H., Miao L., Kollipara R., Horner J.W., DePinho R.A. (2003). Suppression of ovarian follicle activation in mice by the transcription factor foxo3a. Science.

[103] John G.B., Shirley L.J., Gallardo T.D., Castrillon D.H. (2007). Specificity of the requirement for foxo3 in primordial follicle activation. Reproduction.

[104] Pelosi E., Omari S., Michel M., Ding J., Amano T., Forabosco A., Schlessinger D., Ottolenghi C. (2013). Constitutively active foxo3 in oocytes preserves ovarian reserve in mice. Nat. Commun..

[105] Hunt C.R., Gupta A., Horikoshi N., Pandita T.K. (2012). Does pten loss impair DNA double-strand break repair by homologous recombination?. Clin. Cancer Res..

[106] Liu P., Gan W., Guo C., Xie A., Gao D., Guo J., Zhang J., Willis N., Su A., Asara J.M. (2015). Akt-mediated phosphorylation of xlf impairs non-homologous end-joining DNA repair. Mol. Cell..

[107] Xu N., Hegarat N., Black E.J., Scott M.T., Hochegger H., Gillespie D.A. (2010). Akt/pkb suppresses DNA damage processing and checkpoint activation in late g2. J. Cell Biol..

[108] Pedram A., Razandi M., Evinger A.J., Lee E., Levin E.R. (2009). Estrogen inhibits atr signaling to cell cycle checkpoints and DNA repair. Mol. Biol. Cell.

[109] Shen W.H., Balajee A.S., Wang J., Wu H., Eng C., Pandolfi P.P., Yin Y. (2007). Essential role for nuclear pten in maintaining chromosomal integrity. Cell.

[110] Thacker J. (2005). The rad51 gene family, genetic instability and cancer. Cancer Lett..

[111] Brunet A., Bonni A., Zigmond M.J., Lin M.Z., Juo P., Hu L.S., Anderson M.J., Arden K.C., Blenis J., Greenberg M.E. (1999). Akt promotes cell survival by phosphorylating and inhibiting a forkhead transcription factor. Cell.

[112] Astle M.V., Hannan K.M., Ng P.Y., Lee R.S., George A.J., Hsu A.K., Haupt Y., Hannan R.D., Pearson R.B. (2012). Akt induces senescence in human cells via mtorc1 and p53 in the absence of DNA damage: Implications for targeting mtor during malignancy. Oncogene.

[113] Kawamura K., Cheng Y., Suzuki N., Deguchi M., Sato Y., Takae S., Ho C.H., Kawamura N., Tamura M., Hashimoto S. (2013). Hippo signaling disruption and akt stimulation of ovarian follicles for infertility treatment. Proc. Natl. Acad. Sci. USA.

[114] Kim S.Y., Ebbert K., Cordeiro M.H., Romero M., Zhu J., Serna V.A., Whelan K.A., Woodruff T.K., Kurita T. (2015). Cell autonomous phosphoinositide 3-kinase activation in oocytes disrupts normal ovarian function through promoting survival and overgrowth of ovarian follicles. Endocrinology.

[115] Kim S.Y., Ebbert K., Cordeiro M.H., Romero M.M., Whelan K.A., Suarez A.A., Woodruff T.K., Kurita T. (2016). Constitutive activation of pi3k in oocyte induces ovarian granulosa cell tumors. Cancer Res..

[116] Smitz J.E., Cortvrindt R.G. (2002). The earliest stages of folliculogenesis in vitro. Reproduction.

[117] Nguyen Q.N., Zerafa N., Liew S.H., Findlay J.K., Hickey M., Hutt K.J., Bezerra M.E.S., Gouveia B.B., Barberino R.S., Menezes V.G. (2019). Cisplatin- and cyclophosphamide-induced primordial follicle depletion is caused by direct damage to oocytes resveratrol promotes in vitro activation of ovine primordial follicles by reducing DNA damage and enhancing granulosa cell proliferation via phosphatidylinositol 3-kinase pathway. Mol. Hum. Reprod..

[118] Soleimani R., Heytens E., Darzynkiewicz Z., Oktay K. (2011). Mechanisms of chemotherapy-induced human ovarian aging: Double strand DNA breaks and microvascular compromise. Aging.

[119] Roness H., Gavish Z., Cohen Y., Meirow D. (2013). Ovarian follicle burnout: A universal phenomenon?. Cell Cycle.

[120] Kalich-Philosoph L., Roness H., Carmely A., Fishel-Bartal M., Ligumsky H., Paglin S., Wolf I., Kanety H., Sredni B., Meirow D. (2013). Cyclophosphamide triggers follicle activation and “burnout”; as101 prevents follicle loss and preserves fertility. Sci. Transl. Med..

[121] Spears N., Lopes F., Stefansdottir A., Rossi V., De Felici M., Anderson R.A., Klinger F.G. (2019). Ovarian damage from chemotherapy and current approaches to its protection. Hum. Reprod. Update.

[122] Govatati S., Kodati V.L., Deenadayal M., Chakravarty B., Shivaji S., Bhanoori M., Yin X., Pavone M.E., Lu Z., Wei J. (2014). Mutations in the pten tumor gene and risk of endometriosis: A case-control study increased activation of the pi3k/akt pathway compromises decidualization of stromal cells from endometriosis. Hum. Reprod..

[123] Madanes D., Bilotas M.A., Baston J.I., Singla J.J., Meresman G.F., Baranao R.I., Ricci A.G., Takeuchi A., Koga K., Satake E. (2019). Pi3k/akt pathway is altered in the endometriosis patient’s endometrium and presents differences according to severity stage endometriosis triggers excessive activation of primordial follicles via pi3k-pten-akt-foxo3 pathway inhibition of pi3k/akt/mtor pathway for the treatment of endometriosis. Gynecol. Endocrinol..

[124] Makker A., Goel M.M., Das V., Agarwal A. (2012). Pi3k-akt-mtor and mapk signaling pathways in polycystic ovarian syndrome, uterine leiomyomas and endometriosis: An update. Gynecol. Endocrinol..

[125] Takeuchi A., Koga K., Satake E., Makabe T., Taguchi A., Miyashita M., Takamura M., Harada M., Hirata T., Hirota Y. (2019). Endometriosis triggers excessive activation of primordial follicles via pi3k-pten-akt-foxo3 pathway inhibition of pi3k/akt/mtor pathway for the treatment of endometriosis. J. Clin. Endocrinol. Metab..

[126] Yin X., Pavone M.E., Lu Z., Wei J., Kim J.J. (2012). Increased activation of the pi3k/akt pathway compromises decidualization of stromal cells from endometriosis. J. Clin. Endocrinol. Metab..

[127] Barra F., Ferro Desideri L., Ferrero S. (2018). Inhibition of pi3k/akt/mtor pathway for the treatment of endometriosis. Br. J. Pharmacol..

[128] Zhang H., Zhao X., Liu S., Li J., Wen Z., Li M. (2010). 17betae2 promotes cell proliferation in endometriosis by decreasing pten via nfkappab-dependent pathway. Mol. Cell. Endocrinol..

[129] Kitajima M., Dolmans M.M., Donnez O., Masuzaki H., Soares M., Donnez J. (2014). Enhanced follicular recruitment and atresia in cortex derived from ovaries with endometriomas. Fertil. Steril..

[130] Choi Y.S., Park J.H., Lee J.H., Yoon J.K., Yun B.H., Park J.H., Seo S.K., Sung H.J., Kim H.S., Cho S. (2018). Association between impairment of DNA double strand break repair and decreased ovarian reserve in patients with endometriosis. Front Endocrinol..

[131] Kacan T., Yildiz C., Baloglu Kacan S., Seker M., Ozer H., Cetin A. (2017). Everolimus as an mtor inhibitor suppresses endometriotic implants: An experimental rat study. Geburtshilfe Frauenheilkd..

[132] Wang Y., Hu Z., Liu Z., Chen R., Peng H., Guo J., Chen X., Zhang H. (2013). Mtor inhibition attenuates DNA damage and apoptosis through autophagy-mediated suppression of creb1. Autophagy.

[133] Zhou L., Xie Y., Li S., Liang Y., Qiu Q., Lin H., Zhang Q. (2017). Rapamycin prevents cyclophosphamide-induced over-activation of primordial follicle pool through pi3k/akt/mtor signaling pathway in vivo. J. Ovarian. Res..

[134] Adhikari D., Risal S., Liu K., Shen Y. (2013). Pharmacological inhibition of mtorc1 prevents over-activation of the primordial follicle pool in response to elevated pi3k signaling. PLoS ONE.

[135] Zhang X.M., Li L., Xu J.J., Wang N., Liu W.J., Lin X.H., Fu Y.C., Luo L.L. (2013). Rapamycin preserves the follicle pool reserve and prolongs the ovarian lifespan of female rats via modulating mtor activation and sirtuin expression. Gene.

[136] Goldman K.N., Chenette D., Arju R., Duncan F.E., Keefe D.L., Grifo J.A., Schneider R.J. (2017). Mtorc1/2 inhibition preserves ovarian function and fertility during genotoxic chemotherapy. Proc. Natl. Acad. Sci. USA.

[137] Wang W., Luo S.M., Ma J.Y., Shen W., Yin S. (2019). Cytotoxicity and DNA damage caused from diazinon exposure by inhibiting the pi3k-akt pathway in porcine ovarian granulosa cells. J. Agric. Food Chem..

[138] Ganesan S., Keating A.F. (2016). Bisphenol a-induced ovotoxicity involves DNA damage induction to which the ovary mounts a protective response indicated by increased expression of proteins involved in DNA repair and xenobiotic biotransformation. Toxicol. Sci..

[139] Sun X., Su Y., He Y., Zhang J., Liu W., Zhang H., Hou Z., Liu J., Li J. (2015). New strategy for in vitro activation of primordial follicles with mtor and pi3k stimulators. Cell Cycle.

[140] Li J., Kawamura K., Cheng Y., Liu S., Klein C., Liu S., Duan E.K., Hsueh A.J. (2010). Activation of dormant ovarian follicles to generate mature eggs. Proc. Natl. Acad. Sci. USA.

[141] Faddy M.J., Gosden R.G., Gougeon A., Richardson S.J., Nelson J.F. (1992). Accelerated disappearance of ovarian follicles in mid-life: Implications for forecasting menopause. Hum. Reprod..

[142] De Bruin J.P., Dorland M., Spek E.R., Posthuma G., van Haaften M., Looman C.W., te Velde E.R. (2004). Age-related changes in the ultrastructure of the resting follicle pool in human ovaries. Biol. Reprod..

[143] Li Q., Geng X., Zheng W., Tang J., Xu B., Shi Q. (2012). Current understanding of ovarian aging. Sci. China Life Sci..

[144] Gougeon A. (1986). Dynamics of follicular growth in the human: A model from preliminary results. Hum. Reprod..

[145] Oktay K., Kim J.Y., Barad D., Babayev S.N. (2010). Association of brca1 mutations with occult primary ovarian insufficiency: A possible explanation for the link between infertility and breast/ovarian cancer risks. J. Clin. Oncol..

[146] Sharan S.K., Pyle A., Coppola V., Babus J., Swaminathan S., Benedict J., Swing D., Martin B.K., Tessarollo L., Evans J.P. (2004). Brca2 deficiency in mice leads to meiotic impairment and infertility. Development.

[147] Weinberg-Shukron A., Rachmiel M., Renbaum P., Gulsuner S., Walsh T., Lobel O., Dreifuss A., Ben-Moshe A., Zeligson S., Segel R. (2018). Essential role of brca2 in ovarian development and function. N. Engl. J. Med..

[148] Day F.R., Ruth K.S., Thompson D.J., Lunetta K.L., Pervjakova N., Chasman D.I., Stolk L., Finucane H.K., Sulem P., Bulik-Sullivan B. (2015). Large-scale genomic analyses link reproductive aging to hypothalamic signaling, breast cancer susceptibility and brca1-mediated DNA repair. Nat. Genet..

[149] Phillips K.A., Collins I.M., Milne R.L., McLachlan S.A., Friedlander M., Hickey M., Stern C., Hopper J.L., Fisher R., Kannemeyer G. (2016). Anti-mullerian hormone serum concentrations of women with germline brca1 or brca2 mutations. Hum. Reprod..

[150] Shi L., Zhang J., Lai Z., Tian Y., Fang L., Wu M., Xiong J., Qin X., Luo A., Wang S. (2016). Long-term moderate oxidative stress decreased ovarian reproductive function by reducing follicle quality and progesterone production. PLoS ONE.

[151] Yang B., Oo T.N., Rizzo V. (2006). Lipid rafts mediate h2o2 prosurvival effects in cultured endothelial cells. FASEB J..

[152] Das S., Chattopadhyay R., Ghosh S., Ghosh S., Goswami S.K., Chakravarty B.N., Chaudhury K. (2006). Reactive oxygen species level in follicular fluid--embryo quality marker in ivf?. Hum. Reprod..

[153] Kitagawa T., Suganuma N., Nawa A., Kikkawa F., Tanaka M., Ozawa T., Tomoda Y. (1993). Rapid accumulation of deleted mitochondrial deoxyribonucleic acid in postmenopausal ovaries. Biol. Reprod..

[154] Keefe D.L., Niven-Fairchild T., Powell S., Buradagunta S. (1995). Mitochondrial deoxyribonucleic acid deletions in oocytes and reproductive aging in women. Fertil. Steril..

[155] Nogueira V., Hay N. (2013). Molecular pathways: Reactive oxygen species homeostasis in cancer cells and implications for cancer therapy. Clin. Cancer Res..

[156] Kitagishi Y., Matsuda S. (2013). Redox regulation of tumor suppressor pten in cancer and aging (review). Int. J. Mol. Med..

[157] Tait I.S., Li Y., Lu J. (2013). Pten, longevity and age-related diseases. Biomedicines.

[158] Grynberg M., Dagher Hayeck B., Papanikolaou E.G., Sifer C., Sermondade N., Sonigo C. (2019). Brca1/2 gene mutations do not affect the capacity of oocytes from breast cancer candidates for fertility preservation to mature in vitro. Hum. Reprod..

[159] Gunnala V., Fields J., Irani M., D’Angelo D., Xu K., Schattman G., Rosenwaks Z. (2019). Brca carriers have similar reproductive potential at baseline to noncarriers: Comparisons in cancer and cancer-free cohorts undergoing fertility preservation. Fertil. Steril..

[160] Derks-Smeets I.A.P., van Tilborg T.C., van Montfoort A., Smits L., Torrance H.L., Meijer-Hoogeveen M., Broekmans F., Dreesen J., Paulussen A.D.C., Tjan-Heijnen V.C.G. (2017). Brca1 mutation carriers have a lower number of mature oocytes after ovarian stimulation for ivf/pgd. J. Assist. Reprod. Genet..

[161] Johnson L., Sammel M.D., Domchek S., Schanne A., Prewitt M., Gracia C. (2017). Antimullerian hormone levels are lower in brca2 mutation carriers. Fertil. Steril..

[162] Van Tilborg T.C., Derks-Smeets I.A., Bos A.M., Oosterwijk J.C., van Golde R.J., de Die-Smulders C.E., van der Kolk L.E., van Zelst-Stams W.A., Velthuizen M.E., Hoek A. (2016). Serum amh levels in healthy women from brca1/2 mutated families: Are they reduced?. Hum. Reprod..

[163] Wang E.T., Pisarska M.D., Bresee C., Chen Y.D., Lester J., Afshar Y., Alexander C., Karlan B.Y. (2014). Brca1 germline mutations may be associated with reduced ovarian reserve. Fertil. Steril..

[164] Finch A., Valentini A., Greenblatt E., Lynch H.T., Ghadirian P., Armel S., Neuhausen S.L., Kim-Sing C., Tung N., Karlan B. (2013). Frequency of premature menopause in women who carry a brca1 or brca2 mutation. Fertil. Steril..

[165] Guglielmino M.R., Santonocito M., Vento M., Ragusa M., Barbagallo D., Borzi P., Casciano I., Banelli B., Barbieri O., Astigiano S. (2011). Tap73 is downregulated in oocytes from women of advanced reproductive age. Cell Cycle.

[166] Pan H., Ma P., Zhu W., Schultz R.M. (2008). Age-associated increase in aneuploidy and changes in gene expression in mouse eggs. Dev. Biol..

[167] Jang H., Na Y., Hong K., Lee S., Moon S., Cho M., Park M., Lee O.H., Chang E.M., Lee D.R. (2017). Synergistic effect of melatonin and ghrelin in preventing cisplatin-induced ovarian damage via regulation of foxo3a phosphorylation and binding to the p27(kip1) promoter in primordial follicles. J. Pineal Res..

[168] Alexandri C., Stratopoulou C.A., Demeestere I. (2019). Answer to controversy: Mir-10a replacement approaches do not offer protection against chemotherapy-induced gonadotoxicity in mouse model. Int. J. Mol. Sci..

[169] Wang Y., Taniguchi T. (2013). Micrornas and DNA damage response: Implications for cancer therapy. Cell Cycle.

[170] Alexandri C., Stamatopoulos B., Rothe F., Bareche Y., Devos M., Demeestere I. (2019). Microrna profiling and identification of let-7a as a target to prevent chemotherapy-induced primordial follicles apoptosis in mouse ovaries. Sci. Rep..

[171] Dolmans M.M., Martinez-Madrid B., Gadisseux E., Guiot Y., Yuan W.Y., Torre A., Camboni A., Van Langendonckt A., Donnez J. (2007). Short-term transplantation of isolated human ovarian follicles and cortical tissue into nude mice. Reproduction.

[172] Gavish Z., Spector I., Peer G., Schlatt S., Wistuba J., Roness H., Meirow D. (2018). Follicle activation is a significant and immediate cause of follicle loss after ovarian tissue transplantation. J. Assist. Reprod. Genet..

